# Comparative Transcriptome Analysis Reveals the Effect of miR156a Overexpression on Mineral Nutrient Homeostasis in *Nicotiana tabacum*

**DOI:** 10.3390/plants12091739

**Published:** 2023-04-23

**Authors:** Wanhong Liu, Xue Ji, Hanping Cao, Chunsong Huo, Linshen He, Xiang Peng, Ya Yang, Fang Yang, Shu Xiong

**Affiliations:** 1 School of Chemistry and Chemical Engineering, Chongqing University of Science and Technology, Chongqing 401331, China; whliu@cqust.edu.cn (W.L.);; 2 Department of Basic Medicine, Chongqing Three Gorges Medical College, Chongqing 404120, China

**Keywords:** *Nicotiana tabacum*, miR156, SPL, transcriptome analysis, mineral nutrients

## Abstract

Mineral nutrition plays an important role in crop growth, yield and quality. MiR156 is a regulatory hub for growth and development. To date, the understanding of miR156-mediated mineral homeostasis is limited. In this study, we overexpressed Nta-miR156a in the tobacco cultivar TN90 and analyzed the effects of miR156 on mineral element homeostasis in tobacco by comparative transcriptome analysis. The results showed that the overexpression of miR156a caused significant morphological changes in transgenic tobacco. Chlorophyll and three anti-resistance markers, proline, total phenolics, and total flavonoids, were altered due to increased miR156 expression levels. Interestingly, the distribution of Cu, Mn, Zn, and Fe in different tissues of transgenic tobacco was disordered compared with that of the wild type. Comparative transcriptome analysis showed that the overexpression of miR156 resulted in 2656 significantly differentially expressed genes. The expression levels of several metal-transport-related genes, such as *NtABC*, *NtZIP*, *NtHMA*, and *NtCAX*, were significantly increased or decreased in transgenic tobacco. These results suggest that miR156 plays an essential role in regulating mineral homeostasis. Our study provides a new perspective for the further study of mineral nutrient homeostasis in plants.

## 1. Introduction

Mineral nutrients play a vital role in plant growth and development and limit crop quality and yield. The acquisition of trace mineral nutrients from cereals or other crops is a major means of addressing the mineral malnutrition of people, especially in developing countries [1]. Therefore, the study of crop mineral homeostasis not only guarantees plant yield and quality but also lays a foundation for nutrition balance in humans. Regardless of nitrogen, phosphorus, and potassium status in soil, the positive effects of mineral nutrients range from 10 to 70% in increasing crop biomass, preventing yield losses, and enhancing crop resistance to biological and abiotic stresses [2]. Iron ions are able to receive or contribute electrons due to their valence transition, which leads to their important role in maintaining the normal function of photosynthesis and the respiratory chain in plants [3]. The typical symptom of iron deficiency in plants is chlorosis, especially in young leaves [4]. Zinc (Zn) is an important component that maintains the catalytic activity of ethanol dehydrogenase (ADH), glutamate dehydrogenase (GDH), and Cu-Zn superoxide dismutase (CSD) [5,6,7]. Its deficiency results in visible symptoms such as stunted growth, smaller leaves, and loss of greenness [8]. Similar to Zn, copper (Cu) is involved in physiological processes as a cofactor of metalloproteinases, including ascorbate oxidase (AO), laccase, and cytochrome oxidase (CO), in plants [9]. Cu deficiency results in stunted growth and reduced plant resistance to disease [10]. Manganese (Mn) acts as a coenzyme or activator of enzymes, which play a key role in photosynthesis, carbohydrate metabolism, the maintenance of ROS balance, and plant secondary metabolite synthesis [11]. Mn deficiency substantially harms photosynthesis in plants [12]. The excessive accumulation of Fe, Zn, Cu, and Mn as trace essential elements causes heavy metal toxicity to plants. The accumulation of high concentrations of heavy metals in plants can directly induce oxidative stress, thus inhibiting enzyme activity and destroying cell components [13]. Therefore, maintaining a proper level of mineral nutrients is one of the keys to ensuring the normal growth and development of plants. The in-depth analysis of the molecular mechanism of mineral homeostasis will further enhance our understanding of the absorption, transport, and compartmentalization of heavy metals in plants.

Plants have evolved elaborate regulatory mechanisms to cope with the depletion or limited availability of mineral nutrients. The maintenance of mineral homeostasis is closely related to the expression of metal transporters, which are regulated by transcription factors and microRNAs (miRNAs) [14,15,16]. The expression level of OsbHLH133 in rice could be induced by iron deficiency, and the overexpression of OsbHLH133 leads to changes in the distribution of iron in different tissues of rice [17]. Both AtSPL7 and AtCITF1 can directly activate the expression of the Cu uptake-related genes *COPT2*, *FRO4*, and *FRO5* to maintain Cu homeostasis in *Arabidopsis* [18]. Additionally, intertwined feedback loops consisting of SPL7 and Cu-responsive miRNAs, including miR398, miR397, miR408, and miR857, have been shown to play an intuitively important role in maintaining Cu pool balance in plants [19]. ZmbHLH105 inhibits the expression of Fe/Mn transporters to avoid excess Mn accumulation in plants, thus conferring Mn tolerance in maize [20]. Plants in zinc homeostasis mainly depend on the transcription factors bZIP19 and bZIP23, which change zinc concentration-sensing cells [21]. Although transcriptome analysis predicted that multiple miRNAs play an important role in maintaining mineral nutrition homeostasis [22,23,24], experimental evidence to elucidate the regulation of metal ion uptake, transport, and distribution by miRNAs is still limited.

Plant miR156 is a highly conserved gene family whose members play important roles in regulating plant development [25], resisting stress [26,27], secondary metabolite synthesis [28], and ROS homeostasis [29]. Additionally, miR156 is widely involved in regulating the uptake, transport, and distribution of heavy metals in plants. For example, the expression of three Cd transporters, AtZIP1, AtZIP2, and AtABCC1, was significantly upregulated in miR156-overexpressing *A. thaliana* [30]. The expression level of miR156 in tobacco was induced by Cd stress, while the expression pattern of *NtSPL4a* was negatively correlated with miR156, suggesting that the miR156-NtSPL4a module might mediate the response of tobacco to Cd stress [31]. An increasing amount of experimental evidence has shown that miR156 is involved in regulating the homeostasis of essential mineral elements in plants. For example, the expression level of miR156 was significantly regulated in common bean (*Phaseolus vulgaris*) under Mn toxicity stress [24]. MiR156 reduces the iron content in plants by inhibiting the expression of the iron uptake protein AtIRT1 [30]. Osa-miR156 is involved in maintaining iron homeostasis in rice [32]. MiR398 is a key factor in maintaining copper homeostasis, especially when plants are deficient in copper [33]. Notably, the miR156-SPL7 module mediates the *Arabidopsis* response to copper deficiency by regulating miR398 expression [34]. Despite numerous studies showing that miR156 is involved in regulating plant mineral homeostasis, its precise molecular mechanism remains unclear. Comparative transcriptome analysis provides valuable clues to reveal the molecular mechanism by which miR156 regulates mineral homeostasis.

Tobacco is the most widely cultivated nonfood cash crop in the world. Moreover, tobacco, as a model plant, has been widely used in the study of mineral nutrient homeostasis. In this study, we compared plant architecture, physiology, and mineral nutrition between Nta-miR156a-overexpressing tobacco and wild-type TN90 lines. Transcriptome analysis was used to analyze the possible molecular mechanism of miR156 in regulating plant mineral homeostasis. These results provide important information for the comprehensive understanding of the biological function of miR156 and provide new insights into the molecular mechanisms regulating the balance of mineral nutrients in plants.

## 2. Results

### 2.1. Overexpression of Nta-miR156a in Tobacco Leads to Significant Changes in Biological Traits

**Figure 1:**
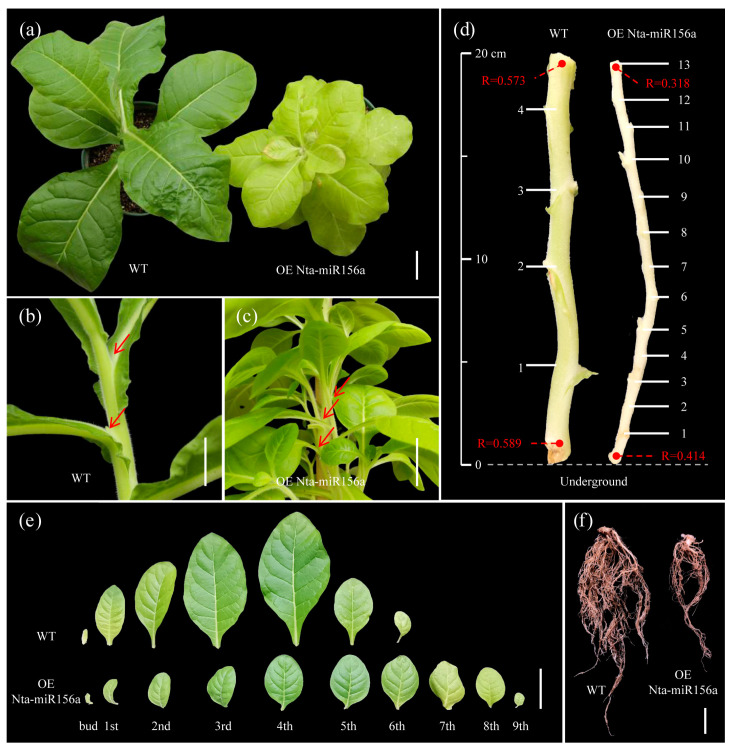
Phenotypic differences between OE Nta-miR156a and WT tobacco. (**a**) Whole view, bar = 5 cm. (**b**,**c**) Partial views of stems of WT and OE Nta-miR156a tobacco, respectively, bar = 5 cm. (**d**) Comparison of the number of lateral branches and node spacing between aboveground 20 cm stem segments of WT and OE Nta-miR156a tobacco. (**e**) Comparison of leaves of WT and OE Nta-miR156a tobacco, bar = 4 cm. (**f**) Comparison of root systems of WT and OE Nta-miR156a tobacco, bar = 3 cm.

Transgenic tobacco was identified by PCR and semiquantitative PCR (Supplementary Figure S1). The overexpression of Nta-miR156a resulted in differences from the WT, mainly in five aspects: the number of leaves, leaf shape, stem diameter, node spacing, and roots (Figure 1). Compared to WT tobacco, transgenic tobacco had more leaves and lateral branches and yellowish leaves (Figure 1a–c). On a portion of the stem segment 20 cm from the ground in the 2-month-old WT and transgenic tobacco, the radii of the bottom and top of the WT stem segment were 0.589 cm and 0.573 cm, respectively, while those of the miR156-overexpressing tobacco were 0.414 cm and 0.318 cm (Figure 1d). The number of their lateral branches and node spacing were significantly different, as shown by the WT plant having four nodes and the tobacco overexpressing miR156 having 13 nodes. Overall, tobacco overexpressing miR156 had more nodes and shorter node spacing. Moreover, the lateral and vertical ratio of leaves of the WT and tobacco overexpressing miR156 was measured separately (Figure S2), and the ratio of the first to the sixth leaf was lower than 0.6 in the WT. In contrast, the tobacco overexpressing miR156 had a value greater than 0.6. Furthermore, the root development of the plants was severely restricted after the overexpression of Nta-miR156a in tobacco (Figure 1f). 

### 2.2. Overexpression of Nta-miR156a Decreased Chlorophyll, Proline, Phenolic Compounds, and Flavonoid Content

**Figure 2:**
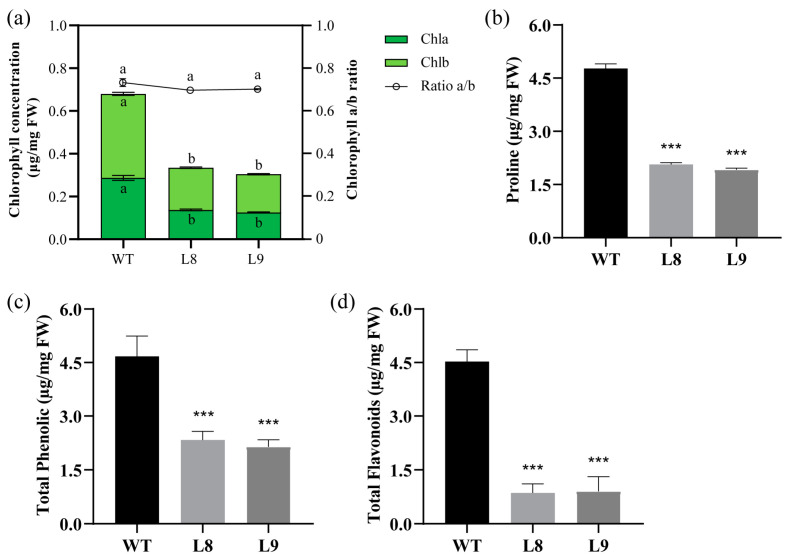
Differences between WT and OE Nta-miR156a tobacco. (**a**) Photosynthetic pigment content. a and b respectively indicate whether the differences between different sample data are statistically significant. The same letter indicates that there is no significant difference between the different samples, while the different letters indicate that there is a significant difference; *p* < 0.01. (**b**) Proline. (**c**) Total phenolics. (**d**) Total flavonoids. Asterisks in the graphs indicate statistical significance of differences calculated by Dunnett’s test method; *** represents *p* < 0.001.

Based on the results of the measurements of plant pigments and essential metabolites, the chlorophyll a and chlorophyll b contents of the miR156 overexpression tobacco (L8 and L9) were significantly lower than those of the WT (Figure 2a), which is consistent with the leaf phenotype observed in Figure 1a. Interestingly, no difference in the chlorophyll a/b ratio was found between the WT tobacco and transgenic tobacco (Figure 2a). To further resolve the intrinsic differences between the transgenic tobacco and WT tobacco, we measured the relative contents of three key metabolic markers (proline, phenolic compounds, and flavonoids). The results showed that the proline content was significantly lower in the miR156 overexpression tobacco (2.07 μg/mg and 1.92 μg/mg) than in the WT tobacco (4.77 μg/mg). In addition, we detected fewer phenolic compounds and flavonoids in the OE lines than in the WT tobacco line (Figure 2b). Specifically, phenolic compounds and flavonoids were reduced 1.00-fold and 4.29-fold, respectively, in the tobacco Nta-miR156a overexpression strain L8 and 1.18-fold and 4.00-fold, respectively, in L9.

### 2.3. Overexpression of Nta-miR156a Disrupts Mineral Nutrient Homeostasis

**Figure 3:**
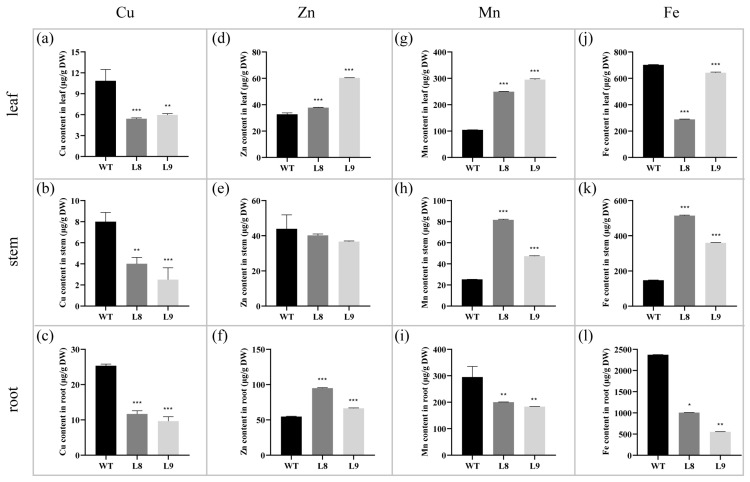
Differences in metal contents in leaf, stem, and root tissues of WT and transgenic tobacco. (**a**–**c**) are Cu contents in leaves, stems, and roots, respectively. (**d**–**f**) are Zn contents in leaves, stems, and roots, respectively. (**g**–**i**) are Mn contents in leaves, stems, and roots, respectively. (**j**–**l**) are Fe contents in leaves, stems, and roots, respectively. Asterisks in the graphs indicate statistical significance of differences calculated by Dunnett’s *t* test method; * represents *p* < 0.05, ** represents *p* < 0.01, and *** represents *p* < 0.001.

To investigate the effects of the overexpression of Nta-miR156a on metal uptake and metal transport in tobacco, we determined the content of four essential metal elements (Cu, Zn, Mn, and Fe) in the leaves, stems, and roots of two kinds of tobacco (Figure 3). In the three tissues of WT tobacco, leaves, stems, and roots, the Cu content was 10.86 μg/g, 8.00 μg/g, and 25.34 μg/g, respectively. The Cu content in the corresponding tissues of the miR156 overexpression tobacco (L8 and L9) was significantly lower than that of the WT (Figure 3a–c). For elemental Zn, significantly higher levels of Zn were found in the leaves and roots of transgenic tobacco than in the WT tobacco (Figure 3d,f), while this was not observed in stems (Figure 3e). Specifically, the Zn content in the leaves and roots of L8 was 1.15 and 1.73 times higher than that of the WT tobacco, respectively. The Zn content in the leaves and roots of L9 was 1.84 and 1.21 times higher than that of WT tobacco, respectively. In addition, the Mn content in the roots of L8 and L9 was lower than that in the WT tobacco (Figure 3i), but the content of Mn in their aboveground parts (leaves and roots) was significantly higher than that in WT tobacco (Figure 3g,h). Unlike the distribution of Zn in tobacco, the Fe content in both the leaves and roots of L8 and L9 was significantly lower than that of the WT tobacco (Figure 3j,l), while the Fe content in stems was 2.49 and 1.44 times higher than that of the WT tobacco, respectively (Figure 3k).

In conclusion, the homeostasis of four essential metal elements (Cu, Zn, Mn, and Fe) was dramatically changed after the overexpression of Nta-miR156a in tobacco. Specifically, we observed a significant decrease in Cu content in the three tissues of transgenic tobacco (leaves, stems, and roots), an increase in Zn accumulation in leaves and roots, the shift of Mn from roots to aboveground parts, and a decrease in Fe accumulation in leaves and roots and its enrichment in stems. 

### 2.4. Changes in the Global Gene Expression Levels of Nta-miR156a-Overexpressing Tobacco

To further elucidate the trait differences between the transgenic tobacco and WT tobacco, we collected leaves for transcriptome sequencing analysis to reveal differences in global gene expression levels caused by miR156 overexpression. For the four samples, WT1, WT2, L8, and L9, 48,983,642, 48,352,528, 56,810,132, and 38,606,522 clean reads were obtained after quality control with an accuracy of 99.9% at 89.51%, 90.90%, 91.73%, and 90.23%, respectively (Table 1). Moreover, 96.22%, 96.72%, 96.71%, and 96.25% of the sequenced reads were accurately aligned to the reference genome. Overall, the data obtained from transcriptome sequencing were quality controlled.

**Figure 4:**
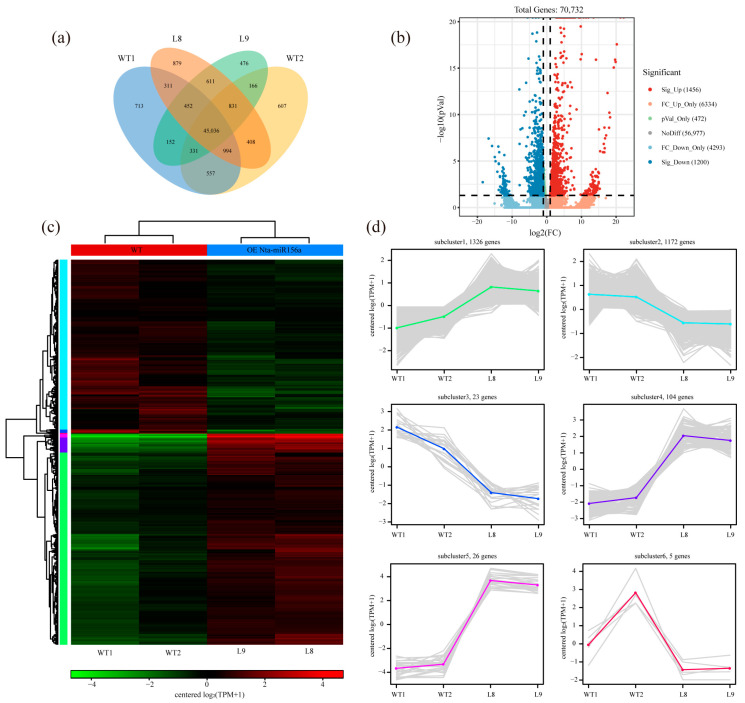
Analysis of gene expression levels between samples. (**a**) Venn diagram of gene expression. (**b**) Volcano map of differentially expressed genes. (**c**) Expression heatmap. (**d**) Expression pattern.

According to the expression of genes in the samples (Figure 4a), approximately 45,036 genes were expressed in all four samples, and they may play a fundamental role in the whole-life activity of tobacco. In addition, 611 genes were only present in L8 and L9, while 557 genes were only detected in the WT tobacco, so this fraction of genes is largely associated with trait differences between the two tobacco types. Genes with a fold change greater than 2 (absolute value of log2 fold change greater than 1) and q value less than 0.05 between the two groups were considered significantly differentially expressed genes. A total of 57,449 genes were not differentially expressed between the two groups, 7790 genes were upregulated, and 5493 genes were downregulated, of which, 1456 genes were significantly upregulated, and 1200 genes were significantly downregulated (Figure 4b). Cluster analysis of the expression of these significantly differentially expressed genes in the four samples (Figure 4a) allowed us to subdivide them into six subclusters (Figure 4c). The majority of these genes clustered into subcluster 1 (1326 genes) and subcluster 2 (1172 genes), and they had low expression differences in both groups. The gene expression levels of subcluster 3 (23 genes), subcluster 4 (104 genes), and subcluster 5 (26 genes) had higher fold changes, while genes in subcluster 6 had higher expression variation within the group (Figure 4d). The 153 genes clustered in subcluster 3, subcluster 4, and subcluster 5 may be the key differentially expressed genes from the perspective of expression change. 

### 2.5. GO and KEGG Enrichment Analysis of Differentially Expressed Genes

**Figure 5:**
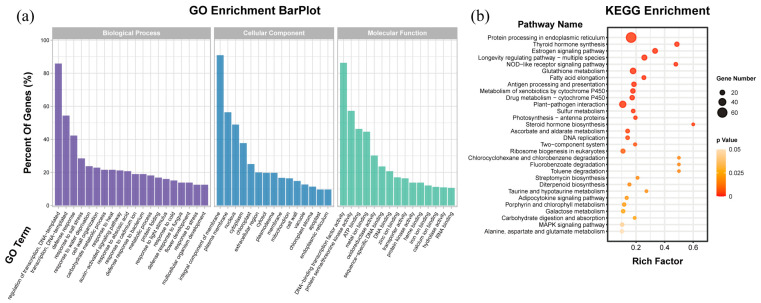
Enrichment analysis of differentially expressed genes. (**a**) GO enrichment analysis. (**b**) KEGG enrichment analysis.

To further analyze the classification and function of differentially expressed genes, we performed an enrichment analysis of differentially expressed genes obtained in the previous step, including GO and KEGG. The results of GO enrichment (Figure 5a) showed that the GO entries enriched in more genes in the classification of biological processes were the regulation of transcription and defense response from a cellular component perspective. The top three GO terms with the highest number of enriched genes were integral components of the membrane, plasma membrane, and nucleus. In addition, according to the GO terms enriched in molecular function, the functional energy classification of differentially expressed genes in the two groups was mainly distinguished in DNA-binding transcription factor activity, protein serine/threonine kinase activity, metal ion binding, oxidoreductase activity, and transporter activity. Interestingly, the GO analysis also showed several terms related to metal ions, such as response to cadmium, zinc ion binding, and iron ion binding. These results suggested that miR156 plays a key role in maintaining mineral nutrient homeostasis in plants.

In the KEGG enrichment results (Figure 5b), the top 20 metabolic pathways with high gene enrichment included protein processing in the endoplasmic reticulum, the NOD-like receptor signaling pathway, glutathione metabolism, sulfur metabolism, photosynthesis, ascorbate and alternate metabolism, porphyrin and chlorophyll metabolism, and the MAPK signaling pathway. The expression levels of two HSP70 genes (LOC107796657 and LOC107796657) associated with the MAPK signaling pathway were significantly increased in miR156-overexpressing tobacco. Multiple glutathione-S-transferase gene expression levels were upregulated in transgenic tobacco, which suggested that miR156 overexpression activates the glutathione metabolic pathway.

### 2.6. Comparative Analysis of Differentially Expressed Genes

**Figure 6:**
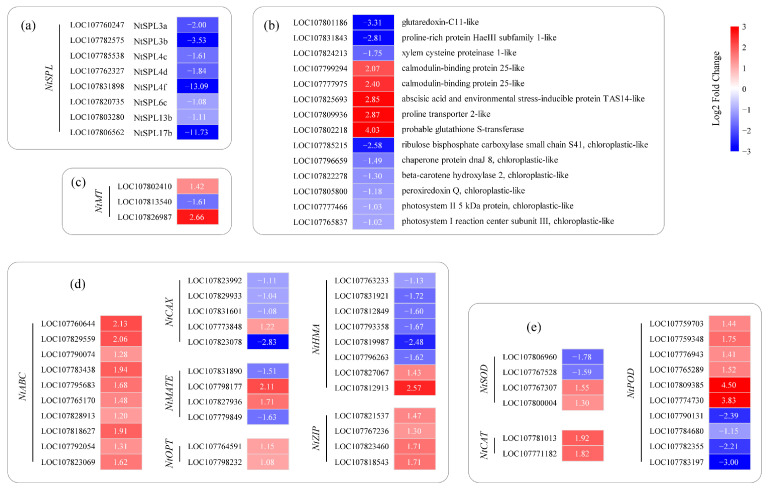
Key differentially expressed genes. Heatmap based on differential TPM values of genes in WT and OE Nta-miR156a tobacco taken as log2 values; red and blue squares indicate higher and lower gene expression in OE Nta-miR156a tobacco than in WT tobacco, respectively. (**a**) NtSPL genes regulated by Nta-miR156a. (**b**) Possible downstream genes regulated by the NtSPL transcription factor. (**c**) NtMT genes are involved in intracellular heavy metal chelation in plants. (**d**) Genes involved in intracellular heavy metal transport in plants. (**e**) Genes related to the plant antioxidase system.

To gain insight into the effect of miR156 on gene expression levels, a total of 75 genes from 2656 DEGs were screened to analyze the difference in their expression levels between wild-type and transgenic tobacco plants. The above genes included the NtSPL family genes (Figure 6a), and target genes regulated by NtSPL (Figure 6b). Meanwhile, the expression levels of mineral-nutrient-response-related genes including metal-chelate protein genes (Figure 6c), transporters (Figure 6d), and enzymatic antioxidant genes (Figure 6e) were also analyzed.

After the overexpression of Nta-miR156a in tobacco, NtSPL genes regulated by Nta-miR156a, such as *NtSPL3a*, *NtSPL3b*, *NtSPL4c*, *NtSPL4d*, *NtSPL4f*, *NtSPL6c*, *NtSPL13b*, and *NtSPL17b*, were significantly downregulated (Figure 6a). In addition, the expression of NtSPL genes was extremely low in transgenic tobacco (TPM < 5), indicating that the expression of NtSPL genes in tobacco is strictly regulated by Nta-miR156a. SPL is an important class of transcription factors in plant cells that have important regulatory roles in plant physiological development, hormone response, and other biological processes [31]. Transcription-factor-binding sites in the promoter sequences of differentially expressed genes were identified through the PlantRegMap online website, and 14 downstream genes that may be regulated by NtSPL transcription factors were identified (Figure 6b). Based on genome annotation, these downstream genes could be classified into two categories: those associated with abiotic plant stress and those associated with chlorophyll. Among the genes associated with plant abiotic stresses, the gene encoding glutoxigenin C11-like protein (LOC107801186), the gene encoding proline-rich protein (LOC107831843), and the gene encoding xylem cysteine protease 1-like protein (LOC107824213) were significantly downregulated compared to those of the WT, with their expression downregulated by 9.94-fold, 7.02-fold, and 3.35-fold, respectively. The genes encoding calmodulin-binding protein 25-like protein (LOC107799294 and LOC107777975), abscisic acid and environmental stress-inducible protein TAS14 (LOC107825693), proline transporter 2 (LOC107809936), and glutathione S-transferase (LOC107802218) were significantly upregulated compared to those of the WT, and their expression was downregulated by 4.20-fold, 5.28-fold, 7.19-fold, 7.30-fold, and 16.33-fold, respectively. In addition, all six genes in the classification of chlorophyll-related genes were significantly downregulated. 

In addition to the regulatory pathway of miR156-SPL-genes, plants usually respond to heavy metal stress in the external environment through various strategies, such as chelation by chelator proteins, transport by metal transporter proteins, and reduction by antioxidant defense enzyme systems. Among the 2656 significantly differentially expressed genes, the metallothionein-encoding genes LOC107802410 and LOC107826987 were elevated by 2.68-fold and 6.33-fold, respectively, and LOC107813540 expression was decreased by 3.06-fold (Figure 6c). Their expression changes in miR156-overexpressing tobacco may have an impact on heavy metal tolerance in tobacco. Additionally, the activation or repression of several metal transporter protein-encoding genes in transgenic tobacco was observed (Figure 6d) from the NtABC family (10 members), NtCAX family (5 members), NtMATE family (4 members), NtOPT family (2 members), NtHMA family (8 members), and NtZIP family (4 members). Among these genes, a total of 16 genes encoding metal transporter proteins from the NtABC family, NtOPT family, and NtZIP family were significantly upregulated in expression and actively participated in the ion transport process in transgenic tobacco. In contrast, the expression of the NtCAX family and NtHMA family in transgenic tobacco seems to be different from that of other metal transporter families, with most of the differentially expressed family members significantly downregulated and only a few genes (one NtCAX family member, LOC107773848, and two NtHMA family members, LOC107827067 and LOC107812913) upregulated. For four members of the NtMATE family, LOC107831890 and LOC107779849 were downregulated by 2.85-fold and 3.08-fold, respectively, and LOC107798177 and LOC107827936 were upregulated by 4.32-fold and 3.27-fold, respectively.

In addition, many antioxidant enzyme-encoding genes were differentially expressed in WT and miR156-overexpressing tobacco, including SOD, CAT, and POD (Figure 6e). Two of the SOD genes (LOC107767307 and LOC107800004), two CAT genes (LOC107781013 and LOC107771182), and six POD genes were upregulated in miR156-overexpressing tobacco; two SOD genes (LOC107806960 and LOC107767528) and four POD genes were downregulated in miR156-overexpressing tobacco. Overall, the overexpression of Nta-miR156a caused the activation of the antioxidant defense system in tobacco.

### 2.7. qRT-PCR Validation of RNA-seq Results

**Figure 7:**
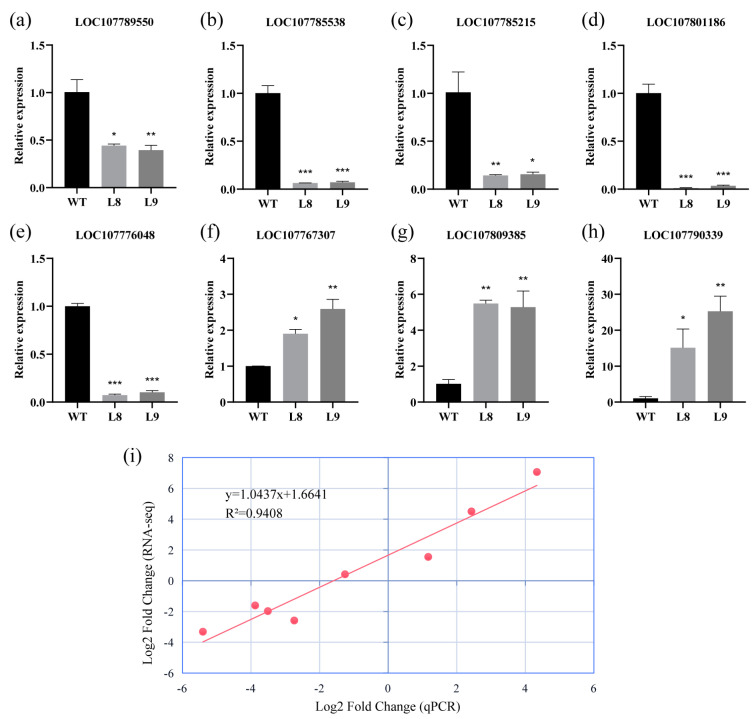
qPCR validation of RNA-seq results. (**a**–**h**) Expression of 8 genes (LOC107789550, LOC107785538, LOC107785215, LOC107801186, LOC107776048, LOC107767307, LOC107809385 and LOC107790339) determined by qPCR. (**i**) Correlation analysis of RNA-seq and qPCR results. Asterisks in the graphs indicate the statistical significance of differences calculated by Dunnett’s t test method; * represents *p* < 0.05, ** represents *p* < 0.01, and *** represents *p* < 0.001.

We verified the relative expression levels of eight transcriptome sequenced genes by qPCR (Figure 7a–h). The results showed that five genes (LOC107789550, LOC107785538, LOC107785215, LOC107801186, and LOC107776048) were significantly downregulated compared to the wild type, and interestingly, the LOC107789550 (NtSPL4a) gene showed no significant expression changes, which was slightly different from the qPCR results. In addition, the other three genes (LOC107767307, LOC107809385, and LOC107790339) showed significantly higher expression levels. We further analyzed the correlation between the two by scatter plots, and the results showed (Figure 7i) that the fitted curve and correlation index between the two were y = 1.0437x + 1.6641 and 0.9408, respectively, which more effectively proved the reliability of the transcriptome data.

## 3. Discussion

Highly conserved miR156 regulates multiple physiological processes in plants. As expected, increased miR156 expression in tobacco altered the expression levels of a large number of genes, including 1456 significantly upregulated genes and 1200 significantly downregulated genes (Figure 4b). As target genes of miR156, at least eight NtSPLs were significantly downregulated in miR156-overexpressing transgenic tobacco (Figure 6a). Subsequently, the disruption of NtSPL expression levels affected several genes or signaling pathways involved in growth and development, stress resistance, and mineral homeostasis.

Plant miR156 acts as a regulatory hub mediating plant developmental processes [35]. A notable phenotype of overexpressed miR156 plants was delayed flowering [36]. In our study, two-year-old miR156-overexpressing tobacco plants cultured in a greenhouse showed no signs of flowering. However, plant flowering is initiated by the highly conserved miR172-AP2 module [37]. The transcription factor SPL could regulate miR172 expression, and the overexpression of the SPL transcription factor induced early flowering in plants [38]. In this study, the overexpression of Nta-miR156a led to the significant downregulation of the expression of at least eight *NtSPL* genes (Figure 6a), which explained the delayed flowering of tobacco overexpressing miR156. These results further indicate that the molecular mechanism by which the miR156-SPL-miR172-AP2 module regulates flowering is highly conserved in the plant kingdom. The overexpression of Nta-miR156 in tobacco could lead to significant changes in plant morphology, including increased lateral branches, shorter internodes and changes in leaf shape (Figure 1). the overexpression of miR156 significantly induced vegetative branch development but inhibited reproductive branch formation (Figure 1b,c). In rice, OsSPL regulates the initiation rate of panicle branching. The dysfunction of SPL caused by miR156 overexpression may lead to transcriptomic reprogramming, which is the key to inducing the proliferation of lateral branches [39]. The overexpression of miR156a led to significant dwarfing in transgenic tobacco, with significantly reduced thicknesses of segment spacing and stem thickness compared with those of the WT (Figure 1d). This finding is consistent with the phenotype in multiple transgenic plants that overexpress miR156 [40,41]. One of the main features of the juvenile to adult transition is an increase in the leaf length-to-width ratio, which is regulated by SPL9 and SPL13 in *Arabidopsis* [42]. The leaf length and width of miR156a-overexpressing tobacco were significantly reduced compared with the controls (Figure S2), suggesting that the leaves of transgenic tobacco were always in a juvenile state under the regulation of miR156. Compared with WT tobacco plants, miR156-overexpressing tobacco plants had fewer lateral roots, which may be related to the Mir-mediated loss of SPL10 function, which decreased the meristem activity of roots [43]. Changes in root system morphology may greatly affect the intake of mineral nutrients.

The overexpression of miR156a significantly changed the photosynthetic pigment content in transgenic tobacco. The concentrations of photosynthetic pigments in transgenic plants were visibly lower than those in WT plants (Figure 2a). The comparative transcriptome results showed that the expression levels of six photosynthetic-system-related genes were decreased in transgenic tobacco overexpressing miR156a (Figure 6b). The overexpression of *VcSPL12* effectively enhanced chlorophyll accumulation by altering the expression levels of several chlorophyll-related genes in blueberry [28], which indicated that SPL was a positively regulated factor involved in chlorophyll biosynthesis in plants. The decrease in chlorophyll content was associated with the downregulation of the expression of eight NtSPLs mediated by miR156a in transgenic tobacco plants (Figure 6a). In addition, the KEGG enrichment results revealed that multiple DEGs were involved in the pathways of photosynthesis and porphyrin and chlorophyll metabolism (Figure 5b). These results suggested that the miR156-SPL module played an important role in regulating chlorophyll synthesis in tobacco.

Moreover, the overexpression of miR156a in tobacco resulted in changes in the content of several nonenzymatic stress response markers, including proline, total phenolics and total flavonoids (Figure 2b–d). Previous studies have shown that miR156 overexpression induces higher proline content in transgenic plants [27,44]. Recently, however, a more detailed study showed that moderately expressed miR156 also resulted in decreased proline content, especially in transgenic plant leaves [45]. The total phenolic content in rejuvenated shoots of transgenic tobacco was lower than that in the WT, which was similar to the results of a previous study [46]. Comparative transcriptome analysis provided some plausible explanations for these changes in stress response markers. For example, the higher expression level of miR156 inhibited the expression of glutaredoxin, which has been shown to be associated with the abiotic stress response and iron homeostasis [47]. Additionally, the proline-rich protein (LOC107831843) is involved in the response to abiotic stress [48], and its expression level was decreased in transgenic tobacco (Figure 6b). Interestingly, miR156 overexpression significantly inhibited the expression level of cysteine proteinase 1, which is an aging marker during tobacco development [49]. These results provide new evidence to confirm the involvement of miR156 in response to stress and plant vegetative phase transition.

There are a variety of metal transporters in plants that function in the uptake, transport, and distribution of mineral elements and play a key role in maintaining the balance of mineral nutrition in plants. In the present study, the distribution of four mineral nutrients, Cu, Zn Mn, and Fe, changed significantly in tissues of the miR156-overexpressing tobacco compared with the WT (Figure 3). This phenomenon is largely because miR156 indirectly/directly regulates the expression of multiple metal transporters, such as NtABC, NtZIP, and NtHMA (Figure 6d). NtPDR3 (pleiotropic drug resistance 3), a member of the ABC transporter family, has been shown to be involved in the regulation of iron homeostasis in tobacco [50]. NtZIP family genes are dedicated to maintaining zinc homeostasis by regulating the root-to-shoot translocation of mineral elements in tobacco plants [51]. In the present study, the expression of several NTABCs and NtZIPs was significantly upregulated in miR156-overexpressing tobacco plants, which was consistent with the results of miR156-overexpressing *A*. *thaliana* [30]. These genes may not be directly regulated by the miR156-SPL module because their promoter regions lack binding sites for SPL transcription factors. Extensive studies in model plant species have shown that HMA is primarily concerned with transporting heavy metals, including Cu and Zn, into cellular compartments or detoxifying heavy metals present in excess concentrations [52,53]. However, not all NtHMA genes showed consistently downregulated expression in transgenic tobacco (Figure 6d). The silencing of multiple NtHMA genes severely hinders pollen development due to Zn deficiency in tobacco [54]. The metal toxicity caused by excessive Mn accumulation in leaves induces the expression of metallothionein in tobacco [55,56]. The upregulated expression of two metallothioneins in transgenic plants may be in response to the excessive accumulation of Mn in the leaves of transgenic tobacco. Additionally, we found that the expression levels of other metal transporters, including NtCAX, NtMATE, and NtOPT, were different between transgenic tobacco and the WT, which fully demonstrated that there is a complex regulatory network which maintains mineral nutrient homeostasis in plants.

**Figure 8:**
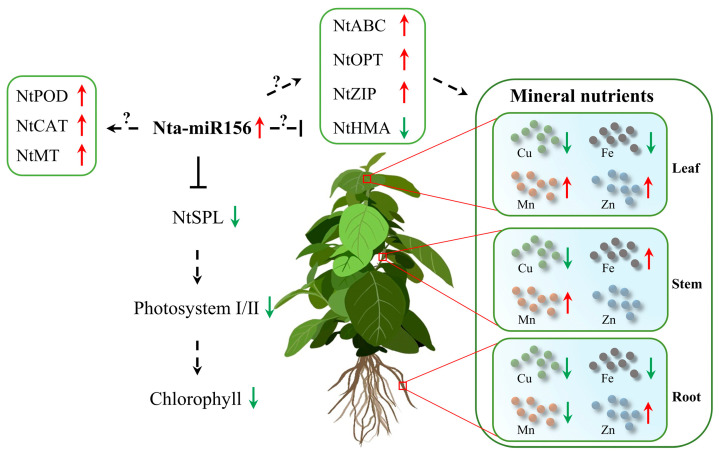
A hypothetical model diagram depicting how miR156 regulates mineral nutrition in tobacco plants. The symbols “↓” and “↑” are used to indicate a decrease or increase in gene expression level or ion content, respectively. A solid line represents a clear conclusion, whereas a dotted line indicates an unclear regulatory mechanism.

In this study, we investigated the impact of miR156 overexpression on mineral nutrition homeostasis in tobacco (Figure 8). The overexpression of miR156 led to a significant reduction in the expression levels of NtSPL transcription factors, resulting in a decrease in chlorophyll content in transgenic tobacco. The expression levels of multiple transporters were altered in miR156-overexpressed tobacco, which significantly affected the distribution of Cu, Fe, Mn, and Zn in tobacco tissues, ultimately leading to the homeostasis of essential mineral nutrients. Furthermore, the overexpression of miR156 indirectly upregulated the level of enzymatic antioxidants, enabling the plant to cope with oxidative stress caused by mineral nutrient imbalance.

## 4. Materials and Methods

### 4.1. Overexpression Vector Construction

Nta-MIR156a-related sequence information was downloaded from the PmiREN 2.0 database (https://pmiren.com/, accessed on 7 May 2021) [57]. Synthetic Nta-MIR156a fragments containing the open reading frame sequences were cloned into the plant expression vector pCXSN under the control of the CaMV35S promoter using the TA cloning method. The recombinant plasmid was named pCXSN::miR156a, which was used for subsequent genetic transformation of tobacco.

### 4.2. Plant Materials and Genetic Transformation

Seeds of tobacco cultivar TN90 were sterilized sequentially by 70% (*v*/*v*) ethanol for 1 min and 20% (*v*/*v*) sodium hypochlorite solution for 30 s and then repeatedly washed with sterile water and sown in MS solid medium. Sterile seedlings were obtained after 2 months of incubation at 25 °C and in 16/8 h (light/dark) conditions. The recombinant plasmid pCXSN::MIR156a was transformed into tobacco according to the Agrobacterium transformation method described by Ana Lilia et al [58]. Specifically, the leaves from disease-free plants were cut into 1 cm^2^ discs and soaked in positively transformed GV301 liquid medium (OD 0.3–0.5) for 8 min; the leaf discs were dried with sterilized filter paper and placed on MS solid medium for coculture in the dark for two days; the leaf discs were transferred to precultural medium (1/2 MS solid medium containing 0.5 mg/L 6-BA, 0.05 mg/L NAA, and 100 mg/L Kan) until calli emerged; healthy calli were transferred to shooting medium (1/2 MS solid medium containing 0.1 mg/L 6-BA, 0.05 mg/L NAA and 100 mg/L Kan) until shoots grew; and the shoots were transferred to rooting medium (1/2 MS containing 0.1 mg/L IBA and 100 mg/L Kan) until roots were generated. The transgenic tobacco seedlings were confirmed by PCR and RT-PCR.

### 4.3. Determination of Stress Markers

The contents of photosynthetic pigments, proline, total phenolic compounds, and total flavonoids in the leaves of WT and miR156 overexpression lines (OE) were assayed according to the Rainbow protocol [59]. Briefly, the plant material (aboveground part) was homogenized into 80% (*v*/*v*) ethanol and centrifuged to precipitate, the absorbance was read at 470 nm, 649 nm, and 664 nm, and the photosynthetic pigment content was calculated using the following Formulas (1) and (2).

The proline content was determined by the ninhydrin colorimetry method. The proline content was determined by ninhydrin colorimetry. The supernatant was fully mixed with 2% (*w*/*v*) ninhydrin reagent, the absorbance was read at 520 nm, and the proline content was calculated according to the L-proline + L-glycine (1:1) standard curve.

The total phenolic compound (TPC) content was determined by the Folin-Ciocalteu method. Briefly, 10% (*v*/*v*) Folin-Ciocalteu reagent was fully mixed with the supernatant, the absorbance was read at 720 nm, and the TPC content was calculated according to the standard curve of gallic acid. 

For total flavonoids (TFLs), the aluminum salt reagent complexation chromogenic method was used to assess samples. The absorbance at 415 nm was read after the samples were thoroughly mixed with 10% (*w*/*v*) aluminum chloride, 1 M potassium acetate and methanol, and the TFL content was calculated according to the standard curve of quercetin.

(1) $$Chla=13.36\times A_{664}\;-\;5.19\times A_{649}$$

(2) $$Chlb=27.43\times A_{649}\;-\;8.12\times A_{664}$$

### 4.4. Metal Content Determination

The roots, stems, and leaves of the samples were dried in an oven at 80 °C for 72 h. After the dried plant material was well ground in a mortar, 20 mg of the powder was weighed and ablated with 5 mL of HNO_3_ on an electric hot plate at 115 °C until the solution was clear and transparent. Then, distilled water was added to the samples to a constant volume of 10 mL, and after appropriate dilutions were made, the Cu, Zn, Fe, and Mn contents of the samples were determined using a flame atomic absorption spectrometer (TAS-986) at 324.8 nm, 213.9 nm, 248.3 nm, and 279.5 nm, respectively [60].

### 4.5. RNA Sequencing

Leaves of 3-month-old wild-type (WT1, WT2) and miR156 overexpression tobacco (L8 and L9) plants were extracted, rapidly frozen in liquid nitrogen, and stored at −80 °C in a freezer. The frozen tobacco tissue samples were transferred to Sangon Biotech (Shanghai) Co., Ltd., for total RNA extraction, and then, the cDNA library was constructed after passing the evaluation by a Qubit fluorometer and Qubit RNA kit. Finally, the cDNA libraries were sequenced on the MGISEQ-2000 (MGI, Tech Co., Ltd., Shenzhen, China) platform. The RNA-seq data generated in this study have been deposited in the Short Read Archive (SRA) database under the accession number PRJNA 953793 (SRR24114095, SRR24114096, SRR24114097, and SRR24114098). 

### 4.6. Sequencing Data Cleaning and Reference Genome Alignment

Trimmomatic software was used to remove splice sequences, remove sequencing reads with sequence lengths less than 35 nt, and filter low-quality (Q value < 20) sequencing reads, and FastQC software was used to count and analyze information such as the quality values of the cleaned data [61]. The TN90 tobacco genome published by the NCBI (https://www.ncbi.nlm.nih.gov/genome/?term=txid4097, accessed on 7 May 2021) was used as the reference genome, and the cleaned sequenced sequences were mapped to the reference genome by HISAT2 software [62].

### 4.7. Expression level Analysis and Enrichment Analysis

The transcript abundance of each gene in the samples was assessed by StringTie software [63], and gene expression in the samples was measured by means of TPM. WT (WT1 and WT2) was defined as Group A, and transgenic tobacco (L8, L9) was defined as Group B. The expression matrices of Groups A and B were analyzed differentially using the DESeq package of R software. Genes whose expression comparison results in Groups A and B simultaneously satisfied q value < 0.05 and |log2 (fold change)| > 1 were considered significantly differentially expressed genes. Volcano maps of differentially expressed genes were drawn by the OmicStudio tool (https://www.omicstudio.cn/tool, accessed on 13 November 2021), and expression clustering analysis was achieved by the gplots package of R software. Gene Ontology (GO) enrichment analysis of differentially expressed genes was performed using the topGO package of R software, and Kyoto Encyclopedia of Genes and Genomes (KEGG) enrichment analysis was achieved by the clusterProfiler package of R software.

### 4.8. Total RNA Isolation and qRT-PCR

RNA was extracted from WT and transgenic tobacco leaves according to the operating instructions of the RNAsimple Total RNA Extraction Kit (Catalog No. DP419) from Tengen (Beijing, China), and the mass and concentration of RNA were identified and measured by agar gel electrophoresis and spectrophotometry. Subsequently, 5 μg of total RNA was reverse transcribed and synthesized into cDNA using the GoScript^TM^ Reverse Transcriptase Kit (Promega, Madison, WI, USA). All cDNA samples were diluted to 10 ng/µL with RNase-free water, and finally, 40 ng of cDNA was used as a template for qPCR. The qPCR mixture was prepared according to the operating instructions of NovoStart^®^ SYBR qPCR SuperMix Plus (Novoprotein, Suzhou, China). The reaction program was set up on the CFX96TM real-time PCR detection system: 95 °C for 1 min; 95 °C for 15 s; 60 °C for 30 s for 45 cycles. qPCR primers for Nta-miR 156 and eight differentially expressed genes were designed by Primer Premier 6.0 software (Table S1), and the expression levels of the eight differentially expressed genes were expressed as NtEF1α (accession number: AF120093) as an internal reference gene for relative quantification [31], and their relative expression was calculated using the 2^-ΔΔCT^ method [64].

### 4.9. Statistical Analysis

In this study, all experimental data were analyzed by one-way ANOVA. Significant differences were analyzed using Dunnett’s multiple range test with GraphPad Prism 8.0 (GraphPad Software, San Diego, CA, United States). Each data point represents the mean ± SD) obtained from three replicates [65].

## 5. Conclusions

In this study, the overexpression of miR156 resulted in significant morphological changes in tobacco, which were specifically manifested as increased lateral branches, shorter internodes, and decreased lateral roots. In addition, the overexpression of miR156 reduced the contents of chlorophyll and three stress-related markers, including proline, and total phenolic and total flavonoid contents. Notably, miR156 plays a key role in regulating mineral nutrient homeostasis. Compared with that of the WT, the distribution of Cu, Zn, Mn, and Fe in the three tissues of transgenic tobacco changed significantly. The transcriptome results showed that the overexpression of miR156 resulted in 2656 DEGs: 1456 upregulated genes and 1200 downregulated genes. Both GO analysis and KEGG analysis showed that these differentially expressed genes were associated with plant development, antioxidant enzyme activity, metal ion binding, metal transporter activity, and plant resistance. To gain a comprehensive understanding of miR156’s role in regulating mineral nutrient balance in tobacco, it is necessary to explore further how reducing miR156 expression affects mineral nutrient homeostasis. In summary, our study provides a new perspective for further research on miR156-mediated mineral nutrient homeostasis in tobacco.

## Figures and Tables

**Table 1:** Summary of RNA-sequencing outcomes.

Sample	WT1	WT2	L8	L9
Raw reads	51,615,914	50,783,586	58,896,570	40,957,114
Clean reads	48,983,642	48,352,528	56,810,132	38,606,522
Raw bases	7,742,387,100	7,617,537,900	8,834,485,500	6,143,567,100

## Data Availability

All data are contained within the article or Supplementary Materials.

## Supplementary Materials

The following supporting information can be downloaded at: https://www.mdpi.com/article/10.3390/plants12091739/s1, Figure S1: Molecular validation of transgenic plants. (a) PCR validation of the inserted fragment, with fragment A representing the Nta-miR156a gene insertions amplified by F-MIR156a and sR-pCXSN-M13, and fragment B representing the hygromycin resistance gene fragments obtained by F-HygR and R-HygR. Panel (b) shows the RT-PCR analysis of miR156 expression levels in transgenic tobacco; Figure S2: The lateral and vertical ratio of leaves; Table S1: Primer list.
