# Supplementary material for: Comparative Transcriptome Analysis Reveals the Effect of miR156a Overexpression on Mineral Nutrient Homeostasis in Nicotiana tabacum
Source: Plants (Basel). 2023 Apr 23;12(9):1739. doi: 10.3390/plants12091739 (PMC10181358; doi:10.3390/plants12091739)
Supplement: Supplementary file 1 [file plants-12-01739-s001.zip › Table S1.pdf]

Table S1. Primer list

Primer name	Primer sequences (5'-3')	Description
RT-U6	GTGCAGGGTCCGAGGTTTTGGACCATTTCTCGAT	RT-U6 stem loop primer
qF-U6	GGAACGATACAGAGAAGATTAGCA	qF-U6 qPCR forward primer
qF-miR156	GGACCTGACAGAAGAGAGT	qF-miR156 qPCR forward primer
qR-miRNA	GTGCAGGGTCCGAGGT	qR-miRNA qPCR reverse primer
RT-miR156	GTCGTATCCAGTGCAGGGTCCGAGGTATTCGCACTGGATACGACGTGCTC	RT-miR156 stem loop primer
F-MIR156a	CAGTGAGCACGCATAGGTTC	Primers for detection of MIR156a insertion fragment
sR-pCXSN-M13	GTTGTAAAACGACGGCCAGT	Primers for pCXSN vector detection
F-HygR	ATCGGACGATTGCGTCGCATC	Hyg^R gene amplification forward primers
R-HygR	GTGTCACGTTGCAAGACCTG	Hyg^R gene amplification reverse primers
qF-NtSPL4a	AGCCATAATAGGGACAGACACT	LOC107789550 expression verification
qR-NtSPL4a	GAGAGCAAGCATATCGTATCCA	
qF-NtSPL4c	AACTTCCACCTCCGTTGAAGAA	LOC107785538 expression verification
qR-NtSPL4c	CCAGTAGTACTAATGGCCGTGT	
qF-NtRuBisCO	CGTCCTCTTTCCCAGTTTCCAG	LOC107785215 expression verification
qR-NtRuBisCO	ATTGCTCCTCGCTCAAATCAGG	
qF-NtGRX	ACTGGCTTTGAGAAGCTTAGGT	LOC107801186 expression verification
qR-NtGRX	AGAGAGCCATCAACATGAAGGG	
qF-NtPCAP1	ACATGGTGGTTGAAGCTGGAGA	LOC107776048 expression verification

qR-NtPCAP1	GCTGTCTCTGTTGCTGGCTCTG	
qF-NtFSD1	CACACCGCTCCTCACCATAGAC	LOC107767307 expression verification
qR-NtFSD1	AGCCTAGAACTGACTGCTTCC	
qF-NtPOD	AGATCCAGTCTTCCGCCCTCT	LOC107809385 expression verification
qR-NtPOD	TGAACAGCTTTCTCACCACACC	
qF-NtHSP	GCGGCAAATTCCTTAGGCGATT	LOC107790339 expression verification
qR-NtHSP	TGGCTTTGACCTCAGGCTTCTT	
