# Supplementary figures and images for: Comparative Transcriptome Analysis Reveals the Effect of miR156a Overexpression on Mineral Nutrient Homeostasis in Nicotiana tabacum
Source: Plants (Basel). 2023 Apr 23;12(9):1739. doi: 10.3390/plants12091739 (PMC10181358; doi:10.3390/plants12091739)

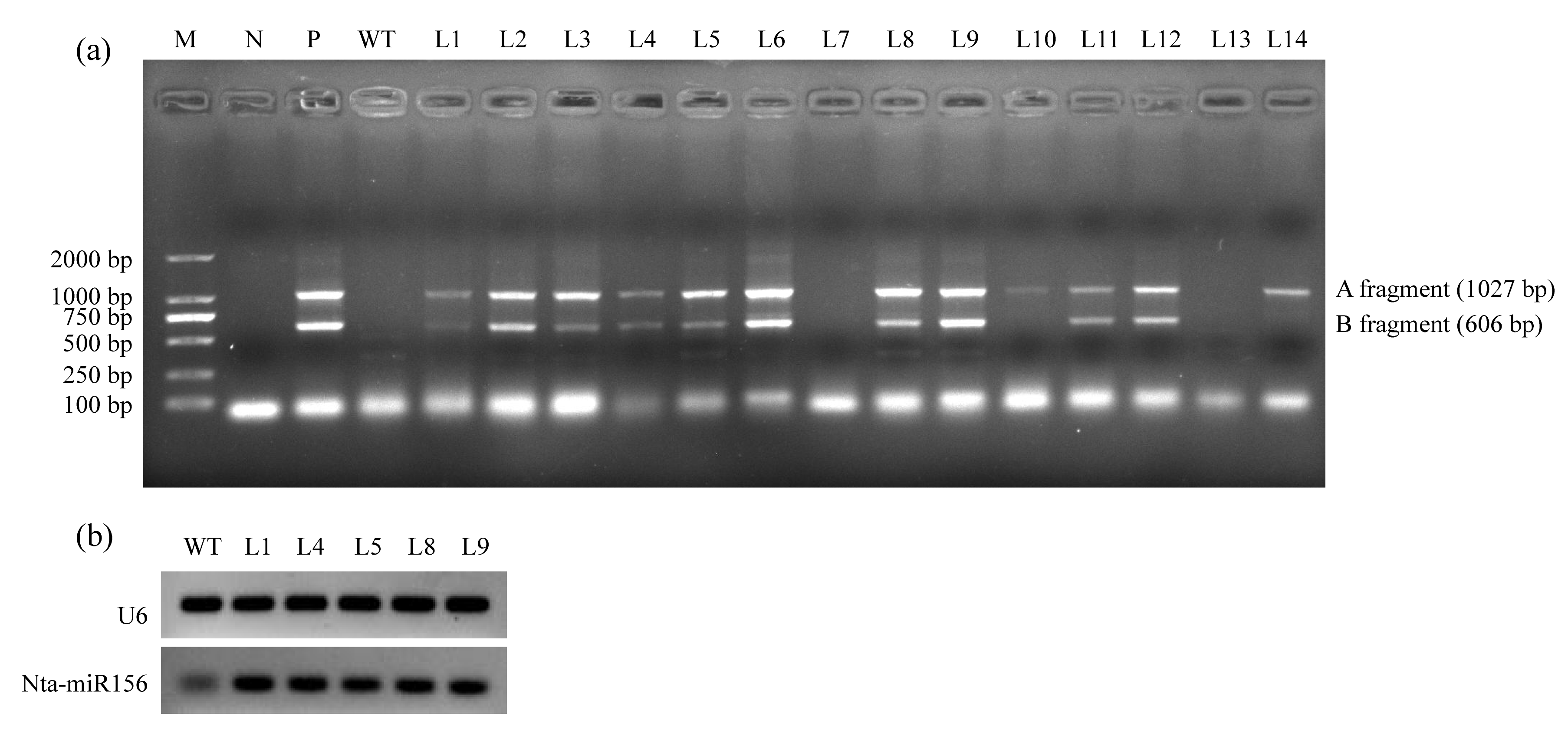

Supplement: Supplementary file 1 [file plants-12-01739-s001.zip › Figure S1.tif]

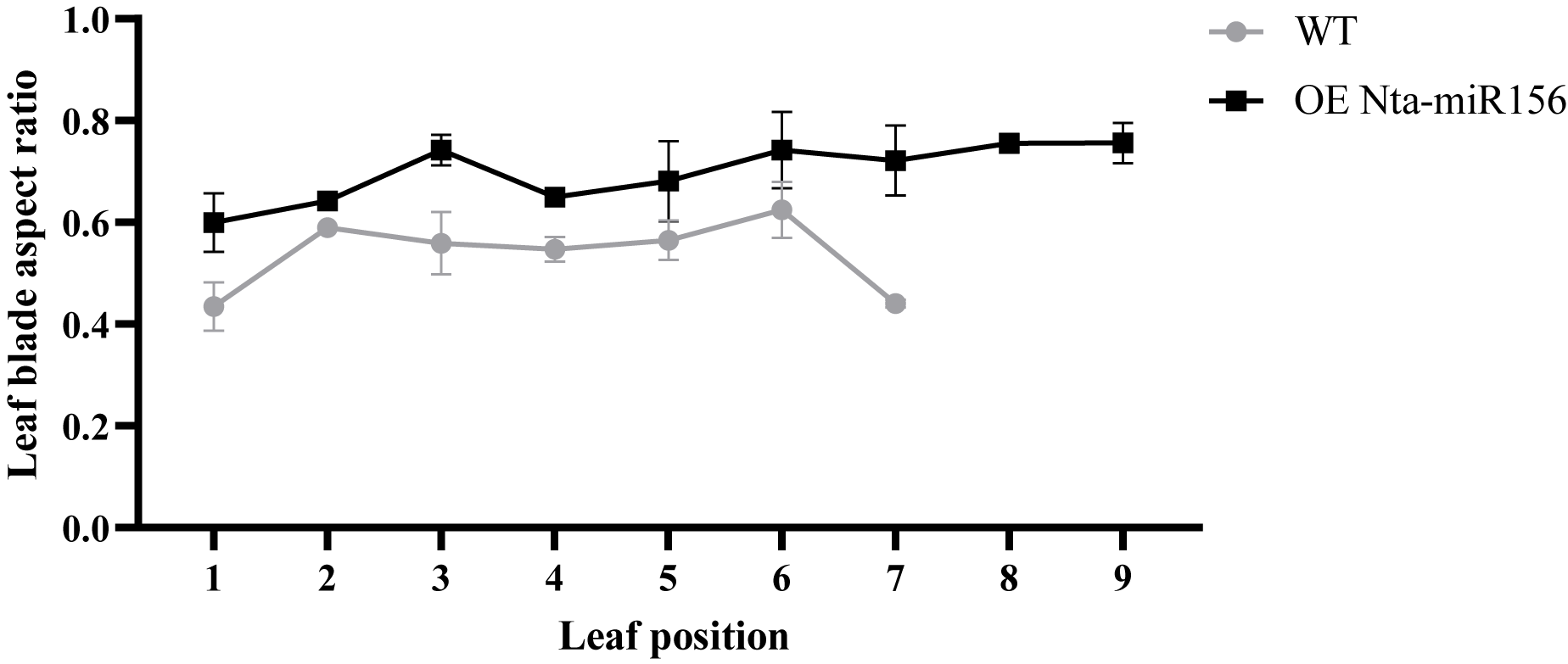

Supplement: Supplementary file 1 [file plants-12-01739-s001.zip › Figure S2.tif]
